# OTME-21. The role of Glioblastoma associated mesenchymal stem cells in immune suppression

**DOI:** 10.1093/noajnl/vdab070.072

**Published:** 2021-07-05

**Authors:** Sanam Sahjram Dharma, Tara Barone, Sheila Figel, Meaghan Birkemeier, Yali Zhang, Robert Fenstermaker, Michael Ciesielski

**Affiliations:** Roswell Park Comprehensive Cancer Center, Buffalo, NY, USA

## Abstract

Glioblastoma (GBM) is an aggressive brain cancer, with an overall survival of 14.6 months. The tumor microenvironment in GBM plays major roles in immunosuppression and modulation of the response to therapies. GBM patients with higher levels of mesenchymal stem like cells (G-MSC) show poor overall survival as compared to patients with no/lower G-MSC levels. Our lab found that levels of G-MSC corelate with CD4+ T cells in humans and murine models of GBM, and with immunosuppressive molecules like PTGS2, the gene for cyclooxygenase 2. To investigate the mechanism by which G-MSCs promote immunosuppression, we isolated G-MSCs from an orthotopic mouse model of GBM and subjected them to RNASeq analysis to obtain an unbiased picture of transcriptomic changes occurring upon activation. We identified changes in multiple immune modulating pathways involving antigen presentation, leukocyte migration and activation, and immune checkpoints. Our findings indicate that G-MSCs represent a key immune modulating faction in the microenvironment. Further dissection of the role of these cells in immune modulation will aid us in understanding the biology of the brain tumor microenvironment and identifying potential combination therapies.

